# Covalently Modified Graphene Oxide and Polymer of Intrinsic Microporosity (PIM-1) in Mixed Matrix Thin-Film Composite Membranes

**DOI:** 10.1186/s11671-018-2771-3

**Published:** 2018-11-12

**Authors:** Elvin M. Aliyev, Muntazim Munir Khan, Afig M. Nabiyev, Rasim M. Alosmanov, Irada A. Bunyad-zadeh, Sergey Shishatskiy, Volkan Filiz

**Affiliations:** 1 Helmholtz-Zentrum Geesthacht, Institute of Polymer Research, Max-Planck-Str. 1, 21502 Geesthacht, Germany; 2 Baku State University, Z. Khalilov str. 23, AZ 1148 Baku, Azerbaijan

**Keywords:** Graphene oxide, Modified graphene oxide, Thin-film composite membrane, Mixed matrix membrane, Gas separation

## Abstract

In this study, mixed matrix membranes (MMMs) consisting of graphene oxide (GO) and functionalized graphene oxide (FGO) incorporated in a polymer of intrinsic microporosity (PIM-1) serving as a polymer matrix have been fabricated by dip-coating method, and their single gas transport properties were investigated. Successfully surface-modified GOs were characterized by Fourier transform infrared spectroscopy (FTIR), UV-Vis spectroscopy, Raman spectroscopy, scanning electron microscopy (SEM), and thermogravimetric analysis (TGA). The effect of FGO loading on MMM morphology and performance was investigated by varying the FGO content in polymer matrix from 9 to 84 wt.%. Use of high FGO content in the polymer matrix helped to reveal difference in interaction of functionalized fillers with PIM-1 and even to discuss the change of FGO stiffness and filler alignment to the membrane surface depending on functional group nature.

**Electronic supplementary material:**

The online version of this article (10.1186/s11671-018-2771-3) contains supplementary material, which is available to authorized users.

## Introduction

Graphene is a promising carbon nanomaterial for future applications. Being a two-dimensional allotropic modification of carbon, graphene can be effectively used as a potential material in molecular separation technologies. Defect-free graphene works as an impermeable material to all molecules. Thanks to the two-dimensional morphology, an incorporation of even small amounts of this material into polymeric membranes helps to effectively hinder the transport of gases and liquids as for example graphene nanoplatelets (less than 0.0075 wt.%) dispersed in PIM-1 reduce the permeability coefficients of gases by a factor of three [1, 2]. In case of PET coated with a low amount of graphene (0.4 wt.%), oxygen permeability was fourfold reduced [3]. APTS-functionalized graphene oxide (GO) incorporated PVDF membrane showed a perfect salt rejection (> 99.9%), while PBI-GO mixed matrix membranes were developed for the organic solvent nanofiltration in the previous works [4, 5]. Current research on thermally rearranged (TR) polymers showed that the incorporation of reduced graphene oxide (rGO) 482 times increased the permeance of CO_2_ while the selectivity of the mixed matrix membrane remained at 35 as it was in pure membrane [6].

Up to now, the research on thin-film composite membrane fabrication has focused on the usage of small amounts of fillers, often 0.1 wt.% of graphene or its derivatives in a polymer [7]. This approach utilizes mostly two-dimensional form of graphene particles and corresponding low percolation threshold [8], at which exfoliated flake-like particles form a continuous phase. Reported pure graphene or graphene oxide membranes contain hundreds of individual layers those serve as a separating factor for molecules [9].

The challenge in achievement of a monolayer graphene is the high energy consumption during fabrication and the interlayer interactions (Van der Waals interactions and π-π stacking) those are causing a strong agglomeration of the graphene sheets. One can overcome this issue by chemical attachment of functional groups, small molecules, and even polymer chains to a graphene sheet via “grafting to” or “grafting from” methods [10].

Graphene oxidation increases the interlayer spacing between the individual sheets and makes them more resistant to agglomeration or stacking. Oxidized graphene contains several oxygen functional groups such as epoxide (C–O–C), phenolic hydroxyl (–OH), and carboxyl (–COOH).

Due to the presence of oxygen functionalities at the edges (–COOH) and in basal plane (C–O–C and –OH), graphene oxide (GO) is well dispersible in different solvents [11, 12]. As a well dispersible material, GO is easy to modify chemically. Chemical functionalization of GO serves as a fascinating mean to increase the affinity of graphene toward various gaseous and liquid substances. Both non-oxidized graphitic domains and oxygen-containing groups of GO can be modified by either non-covalent or covalent functionalization. Non-covalent functionalization involves strong π-π interactions between graphitic domains and functionalizing groups as reported by Dubey et al. [13] and Yang et al. [14].

**Fig. 1:**
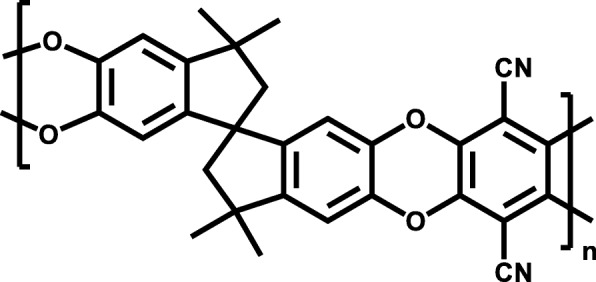
Chemical structure of polymer of intrinsic microporosity (PIM-1)

In this work, we performed covalent chemical functionalization of GO introducing into the graphene structure various functional groups, amines, oximes, modified ferrocenes, thionyl chloride, and phosphorus trichloride, and tried to close a gap by studying polymer/graphene composite materials in a wide range of graphene oxide contents. The research was focused on the single gas transport experiments of three different FGOs incorporated polymer of intrinsic microporosity (PIM-1) (Fig. 1), which is characterized by high gas permeability, but relatively low selectivity. Graphene oxide layers were modified in order to change the nature of the nanoparticles to enhance the single gas transport properties of the PIM-1 membrane. For this purpose, based on possible affinity of functional groups to oxygen, GO was modified with ferrocene derivatives (GO-AEDPPF and GO-dClpf); to carbon dioxide, with amine and oxime derivatives (GO-DMPPA and GO-DClBAO) and to hinder the transport of the large kinetic diameter gas molecules, with phosphorus trichloride (PhChGO). Among these modifications, GO-AEDPPF and GO-DClBAO were chosen for single gas transport experiments as the potential candidates for the enhancement of PIM-1 permeance and selectivity stabilities due to large bulky groups covalently attached onto GO nanoparticle. At the same time, it was intended to observe the effect of high particle loading onto MMM gas transport properties, to compare the membrane performances with state-of-the-art polymer (Table 3 and Additional file 1: Table S1) and graphene-loaded PIM-1 membranes from the gas transport experiments to draw conclusions on the following questions:

- What is the maximum content of a filler, at which selective layer is still working according to the solution-diffusion mechanism;
- Is the alignment of the plate like particles along the surface of the membrane achievable with the dip-coating technique;
- Is there a difference in any property of a filler, which induce breakage of the selective layer;
- Any evidence of membrane affinity toward gases theoretically able to interact with functional groups.

## Methods

### Materials

Graphite (Alfa Aesar, natural, briquetting grade, ~ 100 mesh, 99.9995%), 2,4-dichlorobenzamidoxime (DClBAO, 97%), and tetrahydrofuran (THF) “for synthesis” grade were purchased from Alfa Aeser (Karlsruhe, Germany). Sodium nitrate (NaNO_3_, 99.5%) and *N*,*N*-dimethylformamide (DMF) “for synthesis” grade were purchased from Merck (Darmstadt, Germany), triethylamine (Et_3_N, 99%) from ABCR (Karlsruhe, Germany), H_2_O_2_ (Ph. Eur. Stabilized, 30%) from Roth, 2,5-dimethyl-6-phenylpyrazolo(1a)-pyrimidin-7-amine (DMPPA, 98%), (R_p_)-1-[(1S)-(1-aminoethyl)]-2-(diphenylphosphino) ferrocene (AEDPPF, 97%), 1,1-bisdichlorophosphino-ferrocene (dClpf, ≥ 96%), potassium permanganate (KMnO_4_, 99.0%), sulfuric acid (H_2_SO_4_, 95.0–98.0%), thionyl chloride (SOCl_2_, 99%), phosphorus trichloride (PCl_3_, 99%) and *N*-Methyl-2-pyrrolidone (NMP, > 99%) from Sigma-Aldrich (Steinheim, Germany), and benzene “for synthesis” grade from AppliChem (Darmstadt, Germany). All materials were used as received.

### Synthesis of Graphene Oxide

Graphene oxide was prepared from natural crystalline graphite powder by Hummers method [15]. Thus, 2 g of graphite powder, 1 g of NaNO_3_, and 46 ml of concentrated H_2_SO_4_ were mixed together in a round-bottom flask placed into an ice bath and stirred for 30 min. Then, 6 mg of KMnO_4_ was added into the mixture by portions to prevent the temperature rise above 20 °C and stirred for 2 h. Subsequently, the temperature of suspension was brought to 35 °C and maintained at this level for an hour. Then, 92 ml of distilled water was added at ambient temperature into the brownish gray paste, causing violent effervescence and an increase of temperature to 98 °C. The obtained diluted, brown color suspension was kept at this temperature for several minutes; during this time, the solution changed its color to bright yellow; and after this, the suspension was further diluted with 250 ml of warm distilled water and treated with 20 ml of H_2_O_2_ to reduce the residual permanganate and manganese dioxide. While the suspension was still warm, it was vacuum filtered to avoid precipitation of side products [16]. The filter cake was washed with warm water and centrifuged on a Sigma 6-16 K centrifuge (SciQuip, USA). The obtained sediment was freeze-dried on a Gamma 1-16 LSC plus machine (Martin Christ Gefriertrocknungsanlagen GmbH).

### Synthesis of Functionalized Graphene Oxide (FGO)

#### Synthesis of Chlorinated Graphene Oxide (GO-Cl)

Chlorinated graphene oxide was synthesized according to the procedure reported elsewhere [17]. Briefly, 0.5 g of GO, 10 ml of benzene, and 50 ml of SOCl_2_ were mixed together in a 50-ml round flask and stirred at 70 °C for 24 h. Afterward, the excess of SOCl_2_ was removed by vacuum distillation and the solid was dispersed in acetone. Then, the suspension was filtered, washed twice with acetone, and vacuum dried at 60 °C for 24 h.

#### Synthesis of (R_p_)-1-[(1S)-(1-Aminoethyl)]-2-(Diphenylphosphino)Ferrocene-Modified GO (GO-AEDPPF)

In the presence of 60 ml of DMF, 0.2 g of GO-Cl and 3 ml of triethylamine were allowed to react with 0.02 g of (R_p_)-1-[(1S)-(1-aminoethyl)]-2-(diphenylphosphino)ferrocene (AEDPPF) at 130 °C for 3 days to obtain GO-AEDPPF [18]. After the reaction, the solution was allowed to cool down to ambient temperature and vacuum filtered. The filter cake was washed with DMF, small amount of distilled water (to remove Et_3_N·HCl) and acetone, and vacuum dried at 60 °C for 24 h.

#### Synthesis of 2,4-Dichlorobenzamidoxime-Modified GO (GO-DClBAO)

0.2 g of GO-Cl was dispersed in 50 ml of NMP in a 500-ml round flask. Then, into the suspension, in the presence of 3 ml of trimethylamine, 0.02 g of 2,4-dichlorobenzamidoxime (DClBAO) was added, reaction temperature set at 157 °C and maintained for 72 h to obtain GO-DClBAO. After the reaction, the suspension was filtered under vacuum, washed with NMP, small amount of distilled water (to remove Et_3_N·HCl), and vacuum dried at 40 °C for 24 h.

### Synthesis of Polymer of Intrinsic Microporosity

Polymer of intrinsic microporosity (PIM-1) was synthesized by the route described in the references [19–23]. The synthesized polymer was dried under vacuum at 70 °C for 2 days before being used for characterization and preparation of TFCMs. The molecular weight and polydispersity of PIM-1 were 200 kg/mol and 4–5, respectively, as determined by size exclusion chromatography.

### Thin-Film Composite Membrane Preparation

The thin-film composite membranes with the hybrid selective layer of graphene compounds and PIM-1 were prepared on a microporous polyacrylonitrile (PAN) support (made in-house, average pore size of 22 nm, and 15% surface porosity) [24] using a laboratory scale membrane casting machine [25]. The nanoparticles of GO, GO-AEDPPF, and GO-DClBAO were dispersed in the PIM-1 solution (1 wt.% in THF) at 9, 33, 50, 76, and 84 wt.% loadings with respect to dry polymer weight. Before casting, all solutions were tip sonicated (using Bandelin Sonoplus sonicator) for 1 h.

The selective layer deposition was done by a modified dip-coating method when the porous support at first was brought into the contact with the polymer solution and then rose for 1–2 mm to form a meniscus of the polymer solution between the porous membrane and solution surface. The selective layer was formed at ambient conditions by dragging the polymer solution (at 1.56 m/min speed) out of the meniscus thus achieving uniform, reproducible coating. The evaporation of the solvent was not controlled or influenced. The formed membrane was allowed to dry at ambient conditions.

### Characterizations

Fourier transform infrared (FTIR) spectra were recorded in attenuated total reflectance (ATR) mode on a Bruker ALPHA FT-IR spectrometer (Ettlingen, Germany). The absorbance measurements were done at ambient temperature in a spectral range of 400–4000 cm^−1^ with a resolution of 4 cm^−1^ and average of 64 scans. UV-Vis spectroscopy investigations were carried out on a Specord 210 Plus spectrophotometer (Uberlingen, Germany) in absorbance mode with a 2-nm slit in the wavelength range of 190–1100 nm using integrating sphere. Raman spectra were obtained using a Senterra (Bruker, Ettlingen, Germany) Raman spectrometer with 532-nm excitation laser and 10 fold objective lens. The result was estimated by extracting each single spectrum and the areas corresponding to the D (disorder induced mode, centered around 1300 cm^−1^) and the G bands (graphite mode, around 1550 cm^−1^) have been evaluated by two Gaussian fits. Thermal gravimetric analysis (TGA) was used to investigate the mass loss of functionalized GO samples as a function of temperature. The analysis was carried out on a Netzsch TG209 F1 Iris instrument (Selb, Germany) under argon flow (50 ml min^−1^) from 25 to 900 °C at 10 K min^−1^. A scanning electron microscope Merlin (Zeiss, Oberkochen, Germany) equipped with an energy dispersive X-ray (EDX) analysis system (Oxford, Wiesbaden, Germany) was used to characterize both the surface and cross-sectional morphology of the samples. The samples were fixed on an adhesive, electrically conductive tape, and coated with approx. 6 nm carbon. Secondary electron (SE) images and the EDX spectra were taken at accelerating voltages of 2–3 kV and at 10 kV, respectively. GPC measurement was performed at room temperature in THF on a Waters instrument (Waters GmbH, Eschborn, Germany) using a refractive index detector and polystyrene polymer standards of different molecular weights (Polymer Labs GmbH). Elemental analysis (EA) was carried out with a EuroEA Elementar CHNSO Analyser (EuroVector, Italy). Total carbon, hydrogen, nitrogen, and oxygen were determined by dry combustion method.

After membrane preparation, the samples of 75 mm in diameter were cut and placed into the measurement cell of the membrane testing facility and gas transport properties for CH_4,_ N_2_, O_2_, and CO_2_ were determined at 30 °C and 500 mbar feed pressure. The feed pressure of maximum 500 mbar and permeate pressure of maximum 10 mbar give one the possibility to consider all aforementioned gases as ideal for calculation of membrane permeance. The gas permeation experimental facility is described elsewhere in more details [26]. The membrane permeance (*L*) of a gas can be calculated using the equation:

1 $$ L=\frac{V\bullet 22,41\bullet 3600}{RTAt}\ln \frac{\left({p}_F-{p}_0\right)}{\left({p}_F-{p}_{P(t)}\right)} $$

where *L* is the gas permeance (m^3^(STP) m^−2^ h^−1^ bar^−1^), *V* is the permeate volume (m^3^), 22.41 is molar volume (m^3^(STP) kmol^−1^), 3600 is conversion factor (s h^−1^), *R* is the ideal gas constant (0.08314 m^3^ bar K^−1^ kmol^−1^), *T* is the temperature (K), *t* is the time of measurement between permeate pressure points *p*_*0*_ and *p*_*P(t)*_ (s), *A* is the membrane area (m^2^), and *p*_*F*_*, p*_*0*_, and *p*_*P(t)*_ are the pressures at the feed, permeate side at the start, and at the end time of measurement, respectively (mbar). The ideal selectivity for a gas pair *A* and *B* (α_A/B_) can be calculated by the equation:

2 $$ {\alpha}_{A/B}=\frac{L_A}{L_B} $$

## Results and Discussion

### Synthesis and Characterization of GO and FGO

The synthesis of GO from crystalline natural graphite powder using Hummers method is described in experimental section. In this work, modification of graphene oxide by introduction of various functional groups and properties of the resulting materials are discussed. Synthesized GO renders a non-stoichiometric material that contains different oxygen-containing functional groups, such as phenolic –OH, epoxy (C–O–C), and carboxyl (–COOH) groups those are distributed both at the basal plane and along the edges [27]. GO was chemically modified by introduction of various functional groups.

**Scheme 1:**
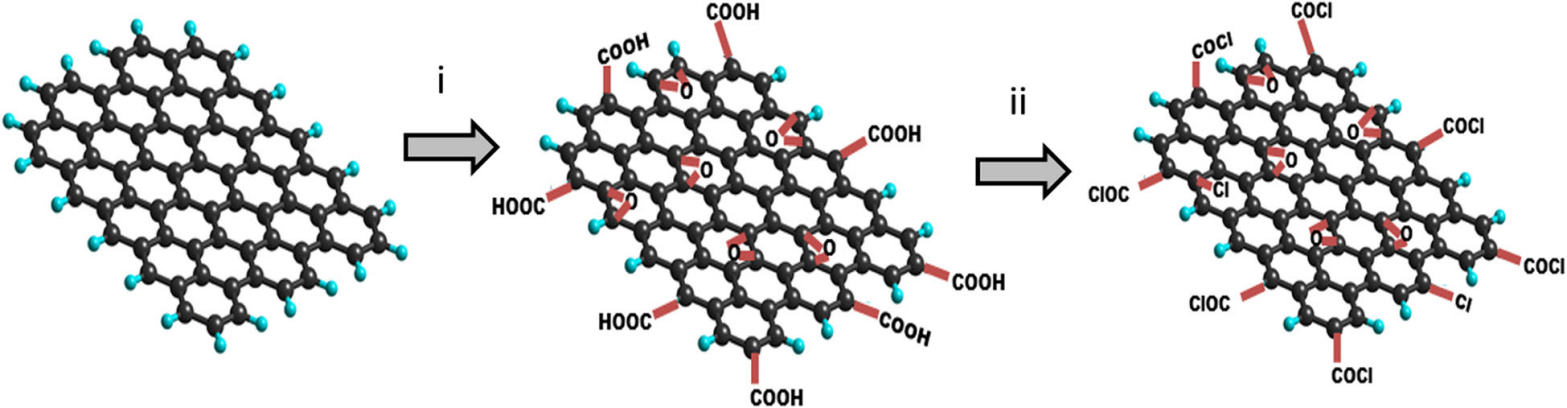
Synthesis of chlorinated graphene oxide (GO-Cl) via Hummers method where (i) KMnO_4_, NaNO_3_, H_2_SO_4_, H_2_O_2_; (ii) SOCl_2_

**Scheme 2:**
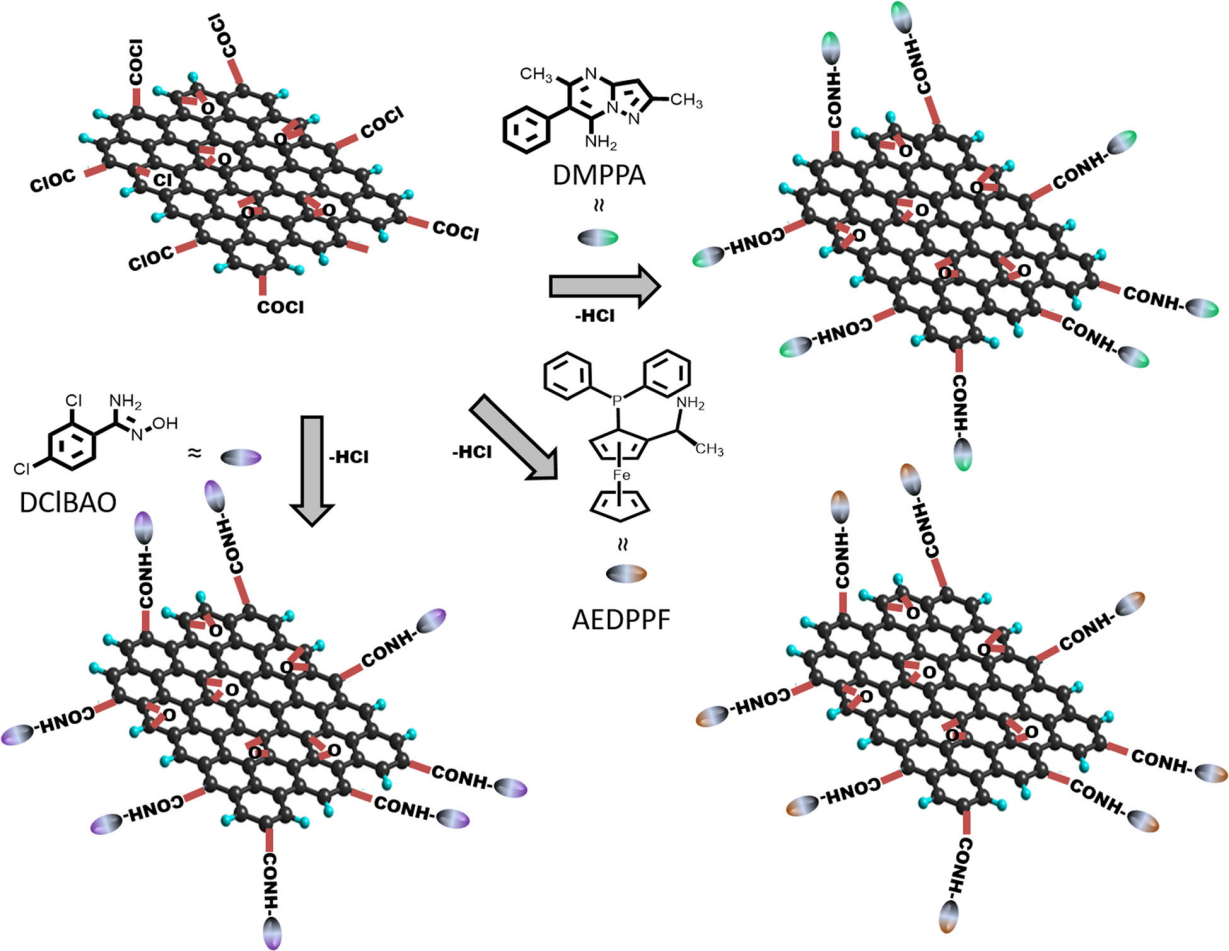
Synthesis of GO-DMPPA, GO-AEDPPF, and GO-DClBAO from GO-Cl

Scheme 1 and Additional file 1: Scheme S1 represent GO synthesis and its modification to GO-Cl, and 1,1-bisdichlorophosphinoferrocene modified graphene oxide (GO-dClpf), and phosphochlorinated graphene oxide (PhChGO), respectively, while Scheme 2 describes GO-Cl modification to GO-DMPPA, GO-DClBAO and GO-AEDPPF with 2,5-dimethyl-6-phenylpyrazolo[1,5-a]-pyrimidin-7-amine, 2,4-dichlorobenzamidoxime and (R_p_)-1-[(1S)-(1-aminoethyl)]-2-(diphenylphosphino)ferrocene, respectively. The synthesis procedure for GO-dClpf, PhChGO and for GO-DMPPA is described in supporting information.

Earlier reports revealed that GO contains oxygen functional groups both in basal planes and at the edges, which can undergo nucleophilic substitution reaction with for example amines [28, 29] and ferrocene [30]. In the present study, nucleophilic substitutions of graphene oxide with modified ferrocene and different amines are discussed.

Elemental composition of graphene oxide and products of its modifications were analyzed by elemental analysis, and the results are listed in the Table 1.

**Table 1:** Elemental analysis of graphene oxide and its modifications

Samples	Element content [wt.]%				C/O ratio	C/H ratio	C/N ratio
	C	H	O	N			
GO	48.1	3.06	45.2	–	1.42	1.32	–
GO-Cl	53.6	2.83	–	–	–	1.59	–
GO-DMPPA	71.5	3.44	17.0	5.73	5.61	1.74	14.6
GO-AEDPPF	70.1	3.69	–	6.27	–	1.58	13.0
GO-DClBAO	71.9	3.26	18.7	5.65	5.12	1.85	14.9
GO-dClpf	48.1	2.93	–	–	–	1.37	–
PhChGO	54.5	2.56	–	–	–	1.79	–

The elemental analysis gives information about bulk composition of the prepared graphene-based samples. As it can be seen from the Table 1, after the synthesis, the C/O ratio for graphene oxide is 1.42 indicating high degree of oxidation, which is accompanied by the largest interlayer spacing [31]. GO-DMPPA and GO-AEDPPF samples have C/O ratio of 5.61 and 5.12, respectively, indicating, together with the reduced oxygen content, successful grafting of amine compounds to GO sheet.

**Fig. 2:**
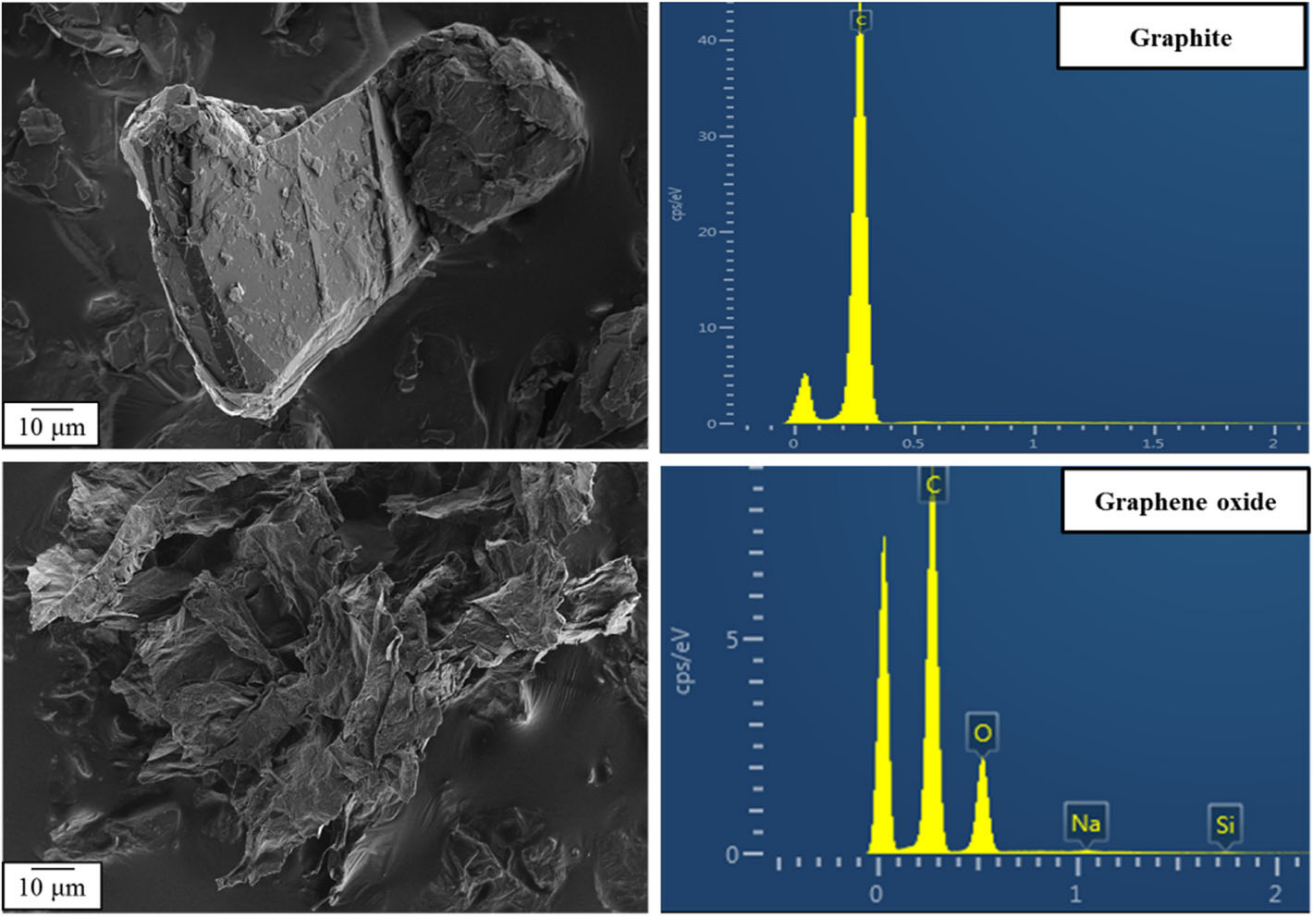
SEM images and EDX spectra show the morphology (left) and the detected elements (right) of graphite (up) and GO particles (down)

Consequently, we investigated graphite and GO by SEM and EDX analysis. The SEM images presented in Fig. 2 show the difference in the graphite morphology before and after the oxidation process.

The EDX analysis (Fig. 2) confirmed the change in the elemental composition of the graphite after its modification to GO. An increase in oxygen amount indicates the successful oxidation process. Other elements in the EDX spectra of chemically modified samples demonstrate grafting of small compounds to graphene sheet (Additional file 1: Figure S1).

**Fig. 3:**
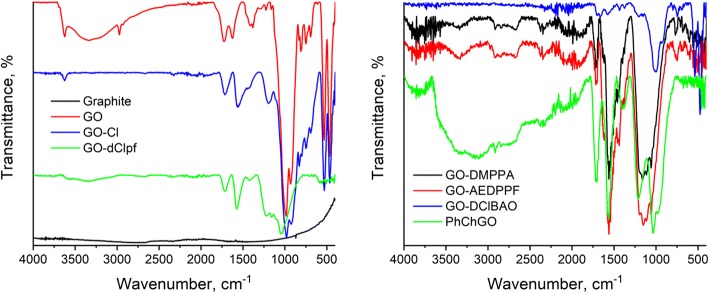
FTIR spectra of GO and its modifications

Chemical modification and successful grafting of different compounds on GO were evaluated by spectroscopic methods such as FTIR, UV-Vis, and Raman spectroscopy. Figure 3 shows the FTIR spectra of GO, chlorinated GO, and chemically modified GO that provides information about chemical interactions between GO and other chemical compounds. The FTIR spectrum of graphite (Fig. 3) appears flat and featureless in the IR region. Pristine GO showed major FTIR stretching vibrations at 3000–3700, 1725, 1628, 1226, and 1055 cm^−1^ corresponding to the intermolecularly bonded –OH stretching vibrations of the hydroxyl group, –C=O stretching (–COOH group), unoxidized graphitic domains, C–O stretching (–COOH group) and C–O–C oxirane stretching (epoxy group) vibrations, respectively (Fig. 3). After thionyl chloride treatment, the carboxylic sites of GO were converted to acid chlorides. This was indicated by peak shifts on FTIR spectrum and almost disappearing of the broad peak at 3000–3700 cm^−1^. Thus, the band representing –C=O stretching vibrations shifts from 1725 to 1717 cm^−1^ indicating the negative inductive effect of the chlorine atom in –COCl group and the presence of quinones [32, 33], which results in vacancy defects [34] formation on GO sheet. A new band at around 1800 cm^−1^ shows the reaction between –COOH and SOCl_2_. Absorbance bands at 1210 cm^−1^ and 717 cm^−1^ describe the C–O stretching vibrations in –COCl group and C–Cl formation in GO, respectively. However, stretching vibrations from C–O–C at 1050 cm^−1^ are still observed.

Compared to pristine GO and GO-Cl, amine and imine-modified GO (GO-DMPPA, GO-AEDPPF and GO-DClBAO) showed the new peaks between 1500 and 1650 cm^−1^ corresponding to reaction products between GO and amine, and imine compounds. Appearance of peaks at around 1560 cm^−1^ show N–H bending vibrations in –CONH group and at 1240 cm^−1^ represent C–N stretching (grafting to aromatic ring). This confirms amide linkages during grafting. A slight broad peak at 3200–3600 cm^−1^ belongs to N–H stretching vibrations, while peaks at 1700, 1717, and 1734 cm^−1^ show the presence of quinones and lactones.

When it comes to GO-dClpf and PhChGO, stretching vibrations for C–O–P group (connected to aromatic rings) are at 1054 cm^−1^. Stretching band at 644 cm^−1^ indicates P–Cl bonding, when peak at 1232 cm^−1^ shows P=O stretching vibration describing the presence of –OH groups in phosphorus moiety.

Additional file 1: Figure S2 depicts the UV-Vis spectrums of GO and its modifications. π-π^*^ transition of carbonyl group was observed between ~ 190 and ~ 200 nm, while n-π^*^ transition of this group can be seen at ~ 265 and ~ 273 nm in GO and its modifications. The absorption peak at around ~ 248 nm attributes to the π-π^*^ transition of C=C bonds from original graphitic structure. The range ~ 295–310 nm observed in UV-Vis measurements is assigned to the n-π^*^ transitions due to presence of C–O–C and C–O–P linkages (Additional file 1: Figure S2).

**Fig. 4:**
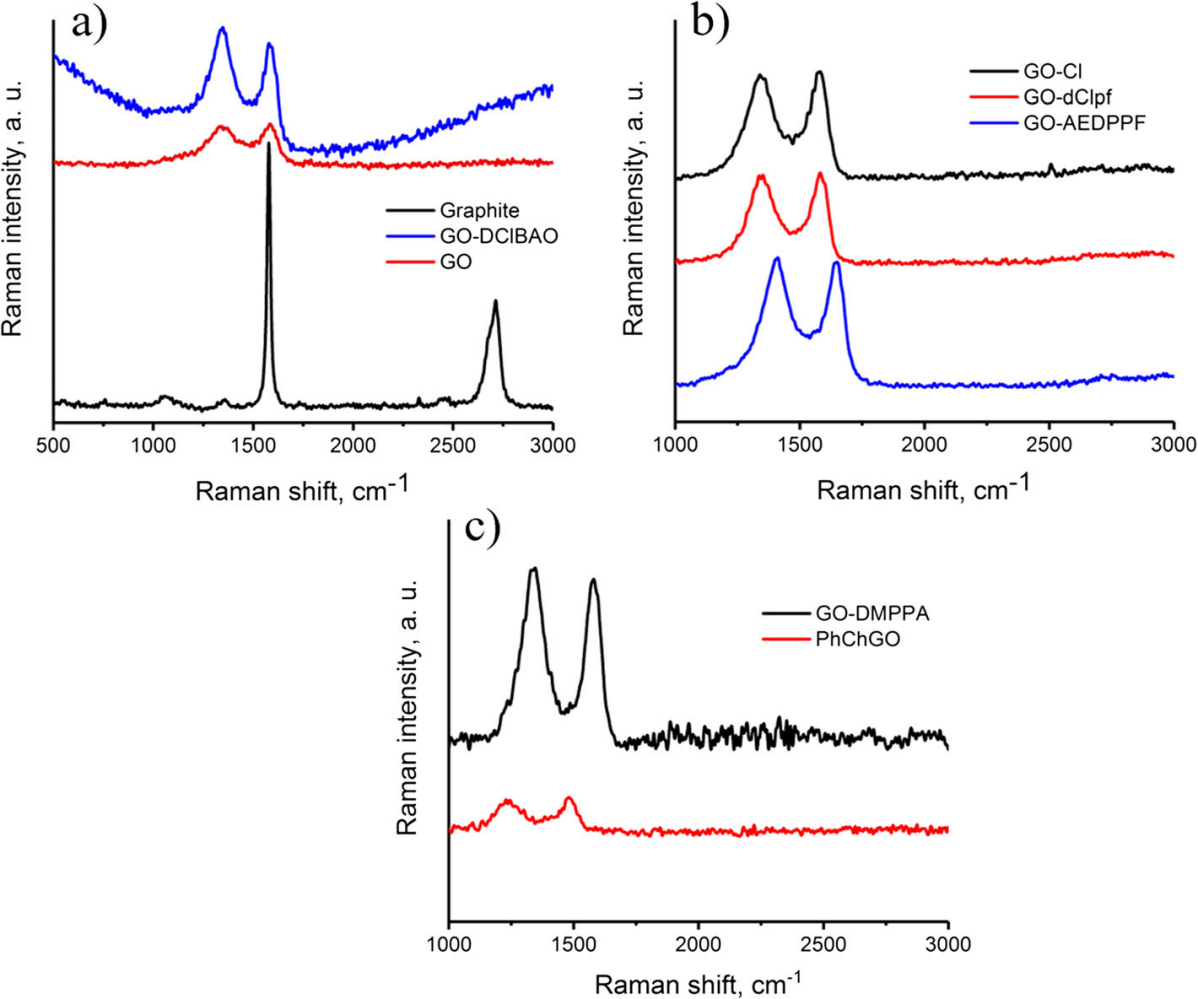
Raman spectra of **a** graphite, GO, and GO-DClBAO; **b** GO-Cl, GO-dClpf, and GO-AEDPPF; and **c** GO-DMPPA and PhChGO

As a powerful technique, Raman spectroscopy was used for the characterization of *sp*^*3*^ and *sp*^*2*^ hybridization of the carbon atoms and examination of ordered vs. disordered crystal structures [35]. The Raman spectra of GO and its modifications displays (Fig. 4) the D-bands at ~ 1340 and ~ 1350 cm^−1^, characteristic Lorentzian G-band at ~ 1580 and ~ 1585 cm^−1^, and 2D-peaks at ~ 2700 cm^−1^. The data are summarized in Table 2.

**Table 2:** Raman spectroscopy results of GO and its modifications

Sample	Raman peaks, cm^−1^			*I*_*D*_/*I*_*G*_
	D-band	G-band	2D-band	
Graphite	1359	1578	2713	–
Graphene oxide	1342	1584	–	~ 0.92
GO-Cl	1340	1580	–	~ 0.96
GO-AEDPPF	1350	1588	–	~ 1.02
GO-DMPPPA	1346	1580	–	~ 1.05
GO-DClBAO	1342	1584	–	~ 1.12
GO-dClpf	1352	1582	–	~ 0.99
PhChGO	1340	1580	–	~ 0.94

D-band is attributed to local defects and disorders, while G-band assigned to the *E*_*2g*_ phonon of carbon *sp*^*2*^ atoms of graphite lattice. 2D-band gives information about the layers of graphene [35]. However, 2D band of monolayer graphene shows a single sharp peak. The ratio of intensities of the D and G bands is often used for determination the number of layers: *I*_*D*_/*I*_*G*_ for all samples was ~ 1, indicating that GO layers are multilayer (Fig. 4).

In order to investigate the thermal stability of GO and its modifications, the thermogravimetric analysis was carried out (Additional file 1: Figure S3). The pristine GO degraded in two main steps. The first step (25–140 °C) by 15% weight loss can be explained by evaporation of absorbed water. The similar behavior is found for GO-Cl with the weight loss of 6%, GO-AEDPPF, 2%; GO-DMPPPA, 1%; GO-DClBAO and GO-dClpf, 7%; and PhChGO—11%. A further major weight loss of about 33% occurs in the temperature range 140–350 °C and corresponds to the decomposition of labile oxygen-containing functionalities. Grafting of amine and imines increased the thermal stability of GO in the temperature range of 200–500 °C [28]. The weight loss for these GO modifications is hovering around 35%. The major weight losses for GO-Cl, GO-dClpf, and PhChGO are seen in the second step: 100–240 °C for GO-Cl by 28%, 120–360 °C for GO-dClpf by 32%, and 150–280 °C for PhChGO by 25%. The lower weight loss below 100 °C of modified GO indicates an enhanced hydrophobicity, which minimizes the amount of absorbed water in comparison with pristine GO.

Additional file 1: Figure S4 shows the SEM images of GO and its modified derivatives. After functionalization, the morphology of graphene (Additional file 1: Figure S4) was changed significantly and it should be mentioned that the morphology of the graphene sheets observed by the SEM is no close to the plane. This observation is important for the formation of a thin-film composite membrane where selective layer is often in the range of 100 nm [36]. In this procedure, graphene oxide layers can wrinkle in the polymer matrix under the shear forces and hinder the gas transport. The SEM images of modified graphene oxide samples show that their morphology is not plane, which gives us information that these modifications can resist the aforementioned forces.

### TFC Membrane Morphology

#### Surface Morphology of the TFC Membranes

In order to evaluate the influence of flake-like fillers having different surface functional groups on the TFC membrane morphology and gas transport performance, the three synthesized graphene-based samples, GO, GO-AEDPPF, and GO-DClBAO, were used as fillers for PIM-1 serving as a matrix polymer of selective layer of the TFC membrane. TFC membranes were produced using PIM-1/GO, PIM-1/GO-AEDPPF, and PIM-1/GO-DClBAO dispersions with different filler to polymer ratio (9 wt.%, 33 wt.%, 50 wt.%, 76 wt.%, and 84 wt.%). The presence of the fillers in PIM-1 solutions changed the color from yellow-green to brown or dark black depending on the fillers’ type. Additional file 1: Figure S5 shows the images of TFC membranes prepared from PIM-1 and PIM-1/GO derivatives.

Additional file 1: Figure S6 shows the SEM surface images of the TFC membranes with the selective layer of pure and filler containing PIM-1. The surface of the pure PIM-1 TFC membrane is smooth and has no features, except one chosen for focusing purposes, which would indicate the presence of defects in the selective layer. An increase in the loading amount of GO and its modifications in PIM-1 changes the appearance of the membrane surface. At 76 wt.% and 84 wt.% solution loadings of GO (Additional file 1: Figure S6e and f) and already at 50 wt.% of GO-AEDPPF (Additional file 1: Figure S6i-k) and GO-DClBAO (Additional file 1: Figure S6n-p) agglomerated GO particles were observed. Since no visible breaks between the polymer and filler particles up to a solution loading of 50 wt.% can be observed one can assume that PIM-1 has a good adhesion to the synthesized graphene oxide materials and that the filler is relatively evenly distributed along the membrane surface. However, to really know about the homogeneity of the filler distribution within the polymer matrix, also the cross section of the membranes was investigated.

#### Cross-sectional Morphology of the Membranes

In Additional file 1: Figure S7, the cross-sectional morphologies of the prepared TFC membranes are shown. The images demonstrate that despite the chemical treatment of the graphene, the particles used for the TFC membrane preparation form agglomerates.

The image of the TFC membrane having pure PIM-1 selective layer demonstrates that the procedure implemented for coating on a porous support gave a uniform layer of polymer with a thickness of approx. 200 nm using a polymer solution with 50 wt.% of concentration. At 9 wt.% of filler in the solution (9 wt.% to GO/polymer composition) GO particles are oriented along the membrane surface, which is expected due to the presence of shear force applied to the forming selective layer during TFC membrane preparation. A competing additional force orienting the particles parallel to the membrane surface may arise from a strong suction of the solvent into the porous support by capillary force resulting in a complete wetting of the porous PAN sublayer. However, due to the high molecular weight of PIM-1, no significant penetration of polymer into the PAN pores was observed, as it can be seen by a border line between continuous polymer layer and porous substrate in the SEM images of pure PIM-1 and PIM-1 with 9 wt.% of GO. At filler concentrations higher than 50 wt.% for GO and 33 wt.% for GO-AEDPPF and GO-DClBAO strong agglomerations of particles were observed with voids within the graphene agglomerates, which were not filled with the polymer. The presence of voids had led to the Knudsen type of gas flow through the membrane at filler loadings of ≥ 50 wt.%. An increase in permeance in case of GO-AEDPPF and GO-DClBAO incorporation can be explained by this phenomenon.

### Gas Transport Performance of Prepared Membranes

Pure gas permeances of TFC membranes for CH_4_, N_2_, O_2_, and CO_2_ with pure PIM-1 and PIM-1 containing FGO such as PIM-1/GO, PIM-1/GO-AEDPPF, and PIM-1/GO-DClBAO were determined at 30 °C on the home-built gas permeation facility. The data of single gas permeance and ideal selectivity according to Eq. 2 were obtained for at least four stamps of the same batch of each TFC membrane; the permeance was calculated as an average value from at least 10 experimental points. The experimental error was determined from the accuracy of the measurement systems permeate volume calibration, from accuracy of pressure sensors, and from the standard deviation of experimental points. The error of the ideal selectivity was taken as a multiplication of experimental errors of corresponding gas permeances (Additional file 1: Table S1). The data for the selectivities and the comparison of the achieved results with the state-of-the-art polymer membranes are presented in Table 3.

**Table 3:** Selectivity for the graphene containing PIM-1 TFC membranes

Membrane code	Filler	Filler content, wt.%	Selectivity				Reference
			O_2_/N_2_	CH_4_/N_2_	CO_2_/N_2_	CO_2_/CH_4_	
PolyActive™	–	–	3.0	4.4	60	14	[38]
Matrimid® 5218	–	–	7.0	1.2	37	30	[39]
Polyetherimide	–	–	7.6	0.58	31	54	[40]
PIM1-0.00096G	Graphene	0.00096	2.6	1.67	14.6	8.8	[1]
PIM-1	–	–	*3.26 ± 0.02*	*1.61 ± 0.01*	*21.3 ± 0.02*	*13.2 ± 0.02*	*This work*
PIM1-9GO	GO	9	*3.06 ± 0.35*	*1.40 ± 0.04*	*18.0 ± 2.82*	*12.9 ± 1.43*	
PIM1-33GO		33	2.09 ± 0.52	1.38 ± 0.08	9.99 ± 4.33	7.24 ± 2.21	
PIM1-50GO		50	0.99 ± 0.01	1.34 ± 0.01	1.32 ± 0.07	0.99 ± 0.04	
PIM1-76GO		76	0.93 ± 0.01	1.35 ± 0.02	0.86 ± 0.01	0.64 ± 0.02	
PIM1-9GO-AEDPPF	GO-AEDPPF	9	*4.70 ± 0.06*	*1.49 ± 0.02*	*25.9 ± 0.35*	*17.4 ± 0.03*	
PIM1-33GO-AEDPPF		33	*3.86 ± 0.14*	*1.49 ± 0.01*	*19.7 ± 1.24*	*13.2 ± 0.02*	
PIM1-50GO-AEDPPF		50	1.44 ± 0.04	1.35 ± 0.01	4.13 ± 0.24	3.06 ± 0.12	
PIM1-76GO-AEDPPF		76	0.95 ± 0.02	1.33 ± 0.005	0.87 ± 0.08	0.65 ± 0.04	
PIM1-9GO-DClBAO	GO-DClBAO	9	*3.74 ± 0.03*	*1.61 ± 0.04*	*21.7 ± 0.15*	*13.5 ± 0.01*	
PIM1-33GO-DClBAO		33	*3.63 ± 0.06*	*1.59 ± 0.05*	*21.1 ± 0.45*	*13.3 ± 0.03*	
PIM1-50GO-DClBAO		50	1.21 ± 0.07	1.36 ± 0.03	2.79 ± 0.54	2.05 ± 0.29	
PIM1-76GO-DClBAO		76	0.71 ± 0.03	1.05 ± 0.09	0.74 ± 0.02	0.7 ± 0.06	

Italicized numbers indicate the selectivity of the membrane equal or exceeding the PIM-1 TFC membrane

**Fig. 5:**
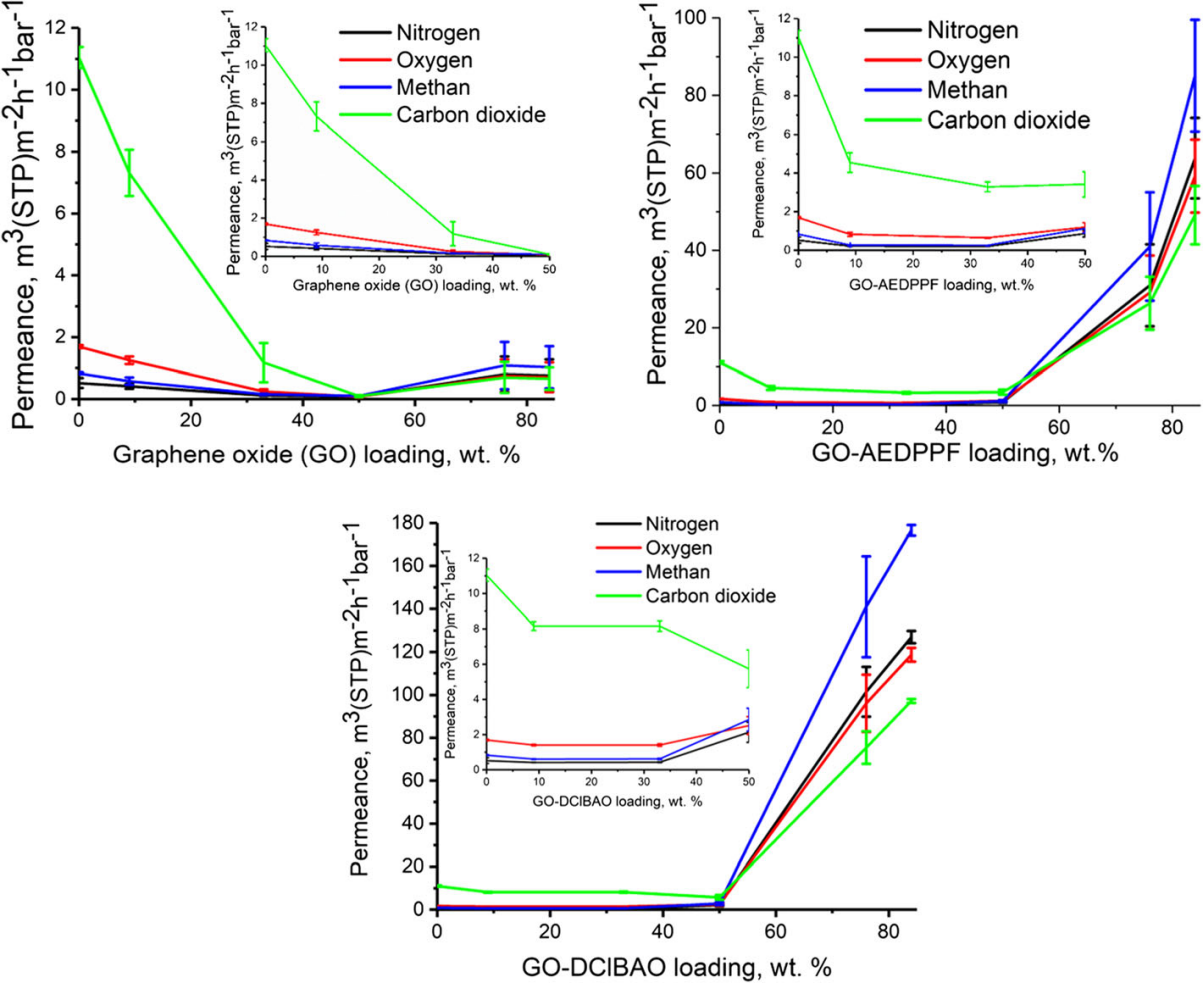
Gas permeances of different gases as a function of GO and its modifications content in PIM-1

Figure 5 demonstrates that the permeance of all gases decreases drastically when graphene-based nanoparticles are incorporated into the selective layer of the TFC membrane. It can be seen that integrity of the selective layer is lost in cases of all three filler materials when the filler concentration exceeds 50 wt.%. At the same time at filler content lower than 50 wt.%, differences in gas transport properties can be observed.

In case of GO containing TFC membranes, due to the presence of flat particles in the PIM-1 oriented along the membrane surface a significant decrease of permeances is observed already at 9 wt.% loading and the most significant permeance loss was observed for CO_2_. The lowering trend is continued until filler loading 50 wt.% and at higher loadings the selective layer become strongly damaged by GO agglomerates, the permeance of all gases increases and the selectivity does not differ from the Knudsen selectivity (Additional file 1: Table S2; Additional file 1: Figure S11).

According to the Additional file 1: Figure S8, which supports Fig. 5, the permeance loss increased in the line CH_4_–O_2_–CO_2_ meaning that “fastest” gases are the most affected by the presence of non-selective graphene based particles. Correspondingly, the selectivity of CO_2_ and O_2_ over N_2_ decreased with the increase of filler content in the polymer.

In comparison to the GO, GO-AEDPPF nanosheets influenced diversely the gas transports properties of MMM. In this case gas permeances were at lowest point at already 33 wt.% loading, remained the same at 50 wt.% and above this filler content the integrity of the selective layer was lost and permeances of all gases increased tremendously due to the presence of non-selective defects as it is shown in Fig. 5.

At 9 wt.% as well as at 33 wt.% loadings of (R_p_)-1-[(1S)-(1-aminoethyl)]-2-(diphenylphosphino) ferrocene modified graphene oxide (GO-AEDPPF), selectivities of carbon dioxide and oxygen over nitrogen were higher than those of pure PIM-1 membrane, which indicates the better interaction of the GO-AEDPPF with PIM-1 (Additional file 1: Figure S9).

It was revealed that 2,4-dichlorobenzamidoxime containing graphene oxide (GO-DClBAO) showed the same performance as it was found in GO-AEDPPF case. The incorporation of 9 wt.% GO-DClBAO decreased gas transport through the PIM-1 based selective layer and it stood at the same level up to 33 wt.% loading (Table 3). The selectivity of CO_2_ and O_2_ over N_2_ was lower at these GO-DClBAO loadings when compared with the results for the GO-AEDPPF containing membranes and was almost the same as of pure PIM-1 membrane (Additional file 1: Figure S10).

An increase in permeance after 33 wt.% loading can be explained by loose aggregation of modified graphene oxide nanosheets those allow to permeate gaseous molecules without any hindrance.

Figure 5 proves that up to 33 wt.% incorporation permeance performance for all gases was leveled off at the same point after 9 wt.% loading. An increase in permeance after 33 wt.% loading an increase for methane, oxygen and nitrogen can be explained by the influence of defects on the modified graphene oxide monolayers. Other increases indicate an aggregation of nanosheets.

**Fig. 6:**
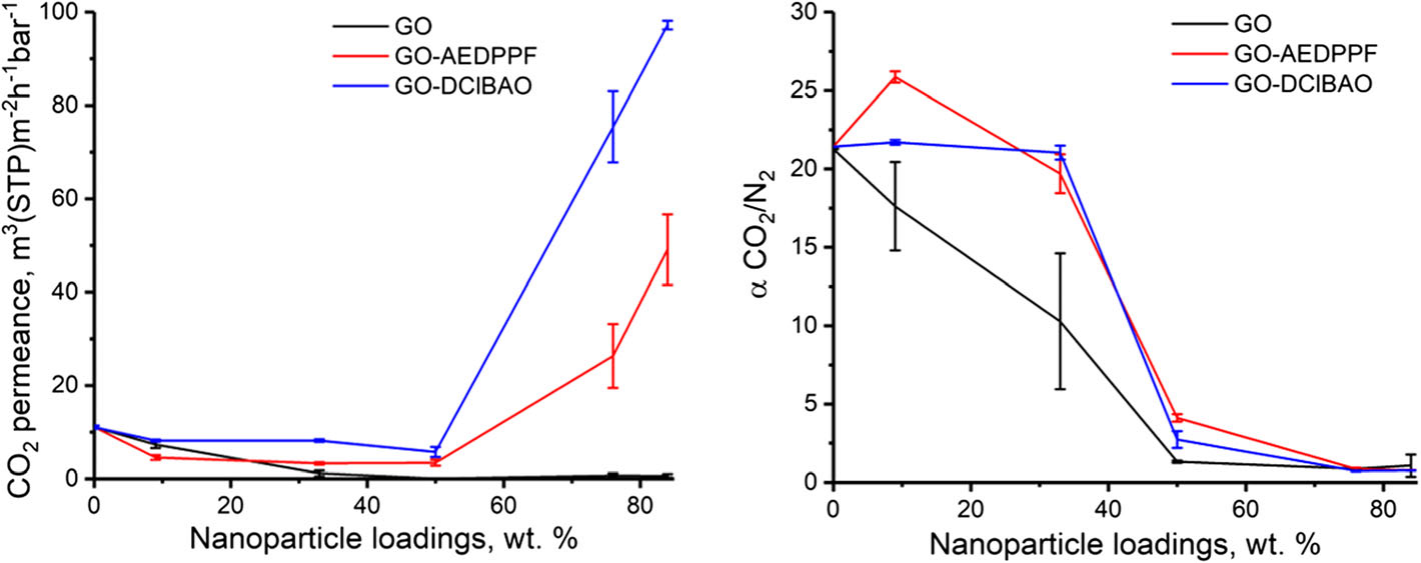
Effect of different FGO incorporation on permeance and selectivity of MMMs

Figure 6 shows the effect of nanoparticle loading on CO_2_ permeance and CO_2_/N_2_ selectivity for three types of MMMs. The effect of the GO modification on the membrane performance when the selective layer is loaded with more than 50 wt.% of filler can be observed. The GO nanoparticles appear to have a strong agglomeration tendency accompanied by the ability of these particles to effectively cover the surface of the membrane, creating an effective barrier for gas transport, when compared to other two compounds resulting in mostly non changing CO_2_ permeance at 50 wt.% and 84 wt.% loadings. The GO-AEDPPF and especially GO-DClBAO containing membranes showed a strong permeance increase at 76 and 84 wt.% loadings indicating properties of the modified GO particles much different from properties of pure GO. The modification of GO with AEDPPF and DClBAO lead to improved particle affinity toward PIM-1 matrix at a particle content below 50 wt.% and to high membrane permeances at 76 and 84 wt.% loadings. Taking into account low permeance of the GO containing PIM-1 TFC membrane at 84 wt.% GO loading and high permeances of both other membranes one can come to the conclusion that modification of the GO with bulky functional groups able to increase polymer-filler compatibility at low filler loadings can change the rigidity of the graphene sheets, which prevents effective alignment of particles along the membrane surface at chosen conditions of TFC membrane preparation. The hypothesis of the particle rigidity change dependent on the functional groups attached to the graphene sheet is to be studied further by e.g. nanoindention method able to characterize mechanical properties of nanometer sized objects [37].

## Conclusions

In this study, different functionalizing agents were used to graft them onto graphene sheets. By EDX and elemental analysis, elemental compositions of the samples are confirmed. Thermogravimetric analysis revealed that the grafting of amines and imines on graphene sheets increased their thermal stability. Raman investigations showed that functionalization leads to multilayer flakes formation. TFC mixed matrix membranes containing PIM-1 as a matrix polymer and three different graphene based fillers synthesized in the course of the current work have demonstrated difference of filler materials properties. The pristine GO acts as an effective barrier material for single gas transport through the PIM-1/GO selective layer with both permeance and ideal selectivity decreasing with an increase of the GO loading. The PIM-1/GO TFC membranes with the filler loading 76 and 84 wt.% have shown low gas permeance indicating that GO can be aligned along the membrane surface under the influence of forces available during the casting solution penetration into the porous support. Gas transport properties of the GO embedded into the PIM-1 matrix are much different from properties of the GO-AEDPPF and GO-DClBAO, which have good compatibility to the PIM-1 at 9 and 33 wt.% loading the MMMs show ideal selectivities overpassing those of the pure PIM-1 TFC membrane. When the loading of these two GO fillers is above 50 wt.% the TFC membranes show significant increase of the permeance compared to GO-PIM-1 MMM, indicating that the matrix PIM-1 polymer and forces available during the membrane formation are not able to effectively align these particles along the membrane surface. The observation of high membrane permeance, which is similar to the permeance of the porous PAN membrane, has lead us to the conclusion that introduction of large amounts of bulky functional groups onto the surface of graphene sheet is leading to increase of the graphene rigidity; this effect is to be studied further with methods able to investigate mechanical properties of nanometer-sized objects.

## Additional file

Additional file 1: Supporting information (DOCX 9050 kb)

## Abbreviations

AEDPPF: (R_p_)-1-[(1S)-(1-Aminoethyl)]-2-(diphenylphosphino) ferrocene
DClBAO: 2,4-Dichlorobenzamidoxime
dClpf: 1,1-Bisdichlorophosphino-ferrocene
DMPPA: 2,5-Dimethyl-6-phenylpyrazolo(1a)-pyrimidin-7-amine
FGO: Functionalized graphene oxide
FTIR: Fourier transform infrared spectroscopy
GO: Graphene oxide
GO-AEDPPF: (R_p_)-1-[(1S)-(1-aminoethyl)]-2-(diphenylphosphino)ferrocene-modified GO
GO-Cl: Chlorinated graphene oxide
GO-DClBAO: 2,4-Dichlorobenzamidoxime-modified GO
GO-dClpf: 1, 1-Bisdichlorophosphinoferrocene-modified GO
GO-DMPPA: 2, 5-Dimethyl-6-phenylpyrayolo [1, 5-a]-pyrimidin-7-amine-modified GO
GPC: Gel permeation chromatography
PhChGO: Phosphochlorinated GO
SEM: Scanning electron microscopy
TFC: Thin-film composite
TGA: Thermal gravimetric analysis
