# Supplementary material for: Covalently Modified Graphene Oxide and Polymer of Intrinsic Microporosity (PIM-1) in Mixed Matrix Thin-Film Composite Membranes
Source: Nanoscale Res Lett. 2018 Nov 12;13:359. doi: 10.1186/s11671-018-2771-3 (PMC6232192; doi:10.1186/s11671-018-2771-3)
Supplement: Supplementary file 1 — Supporting information (DOCX 9050 kb) [file 11671_2018_2771_MOESM1_ESM.docx]

**Covalently modified graphene oxide and polymer of intrinsic microporosity (PIM-1) in mixed matrix thin film composite membranes**

**Supporting Information**

Elvin M. Aliyev1,2, Muntazim Munir Khan^[[1]](#footnote-1)^1, Afig M. Nabiyev^[[2]](#footnote-2)^2, Rasim M. Alosmanov^[[3]](#footnote-3)^2, Irada A. Bunyad-zadeh^[[4]](#footnote-4)^2, Sergey Shishatskiy^[[5]](#footnote-5)^1, Volkan Filiz^[[6]](#footnote-6)^1^,^ ^[[7]](#footnote-7)^

**Methods**

**Synthesis of Functionalized Graphene Oxide (FGO)**

***Synthesis of 2, 5-Dimethyl-6-Phenylpyrayolo [1, 5-a]-Pyrimidin-7-Amine (GO-DMPPA)***

In the presence of 60 ml of DMF, 0.2 g of GO-Cl and 3 ml of triethylamine were allowed to react with 0.054 g of 2,5-dimethyl-6-phenylpyrazolo[1,5-a]-pyrimidin-7-amine (DMPPA) at 130 °C for 3 days to obtain GO-DMPPA. After the reaction, the solution was allowed to cool down to ambient temperature and vacuum filtered. The filter cake was washed with DMF, small amount of distilled water (to remove Et_3_N·HCl) and acetone, and vacuum dried at 60 °C for 24 hrs.

***Synthesis of 1, 1-Bisdichlorophosphinoferrocene Modified GO (GO-dClpf)***

In a 500 ml round flask 0.2 g GO was dispersed in 20 ml of benzene. Then 0.02 g of 1, 1-bisdichlorophosphinoferrocene *(dClpf)* was added into the suspension and the mixture was heated to 70 °C for 24 hrs. in an oil bath. Afterwards, the suspension was cooled to room temperature and filtered under vacuum to obtain brownish black precipitate of GO-dClpf, and vacuum dried at 60 °C for 24 hrs.

***Synthesis of Phosphochlorinated GO (PhChGO****)*

0.5 g of GO was dispersed in 10 ml of chloroform in a 50 ml triple-neck round bottom flask. 4 ml of PCl_3_ (phosphorus trichloride) was added by portions in order to keep the temperature stable, and the mixture was refluxed for 3 hours at ambient temperature under oxygen flow (8 L/h). The final sediment was washed with distilled water up to pH 7, vacuum filtered and dried in vacuum at 50 °C, 24hrs.

**Results and Discussion**

**
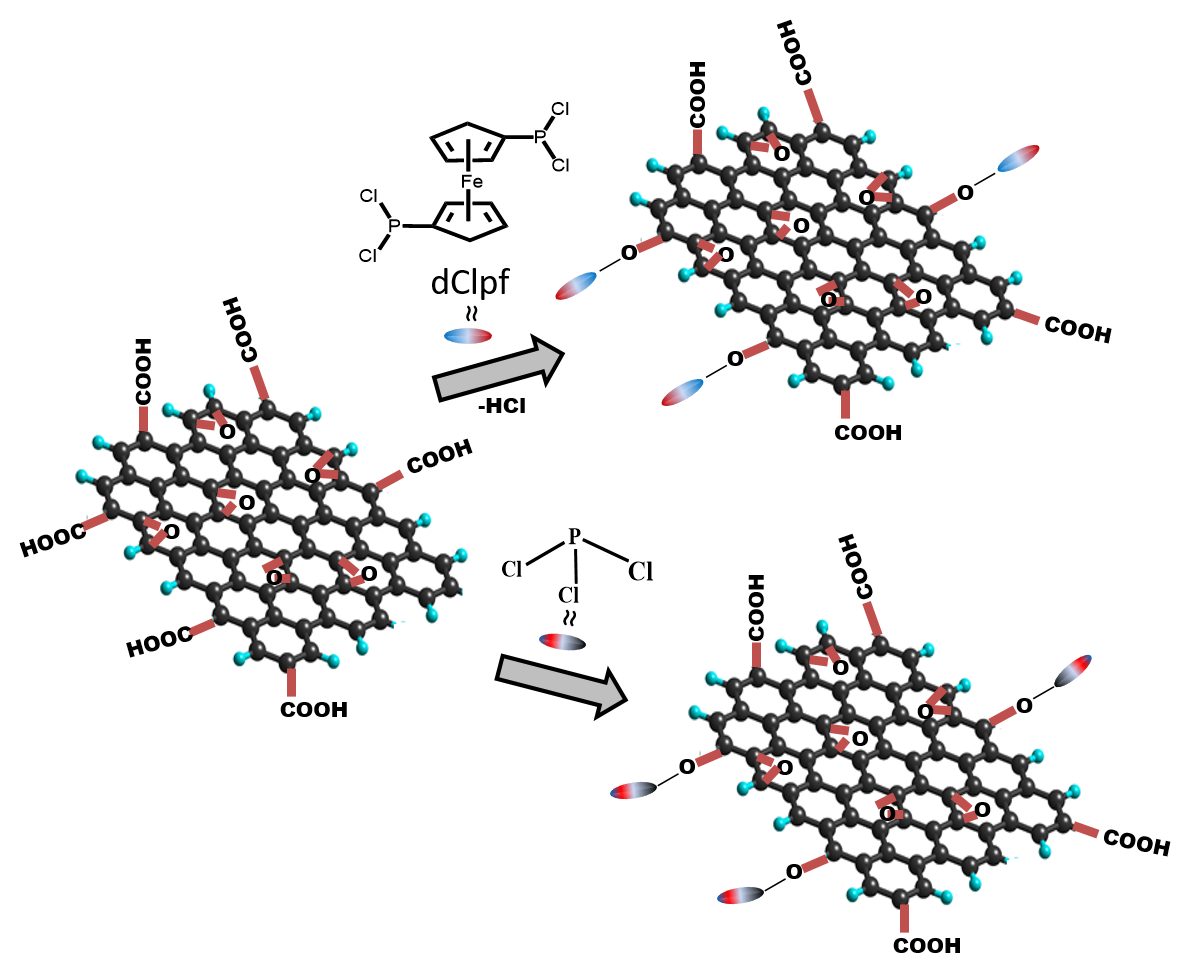
**

**Scheme 1SI. Synthesis of GO-dClpf and PhChGO from GO.**


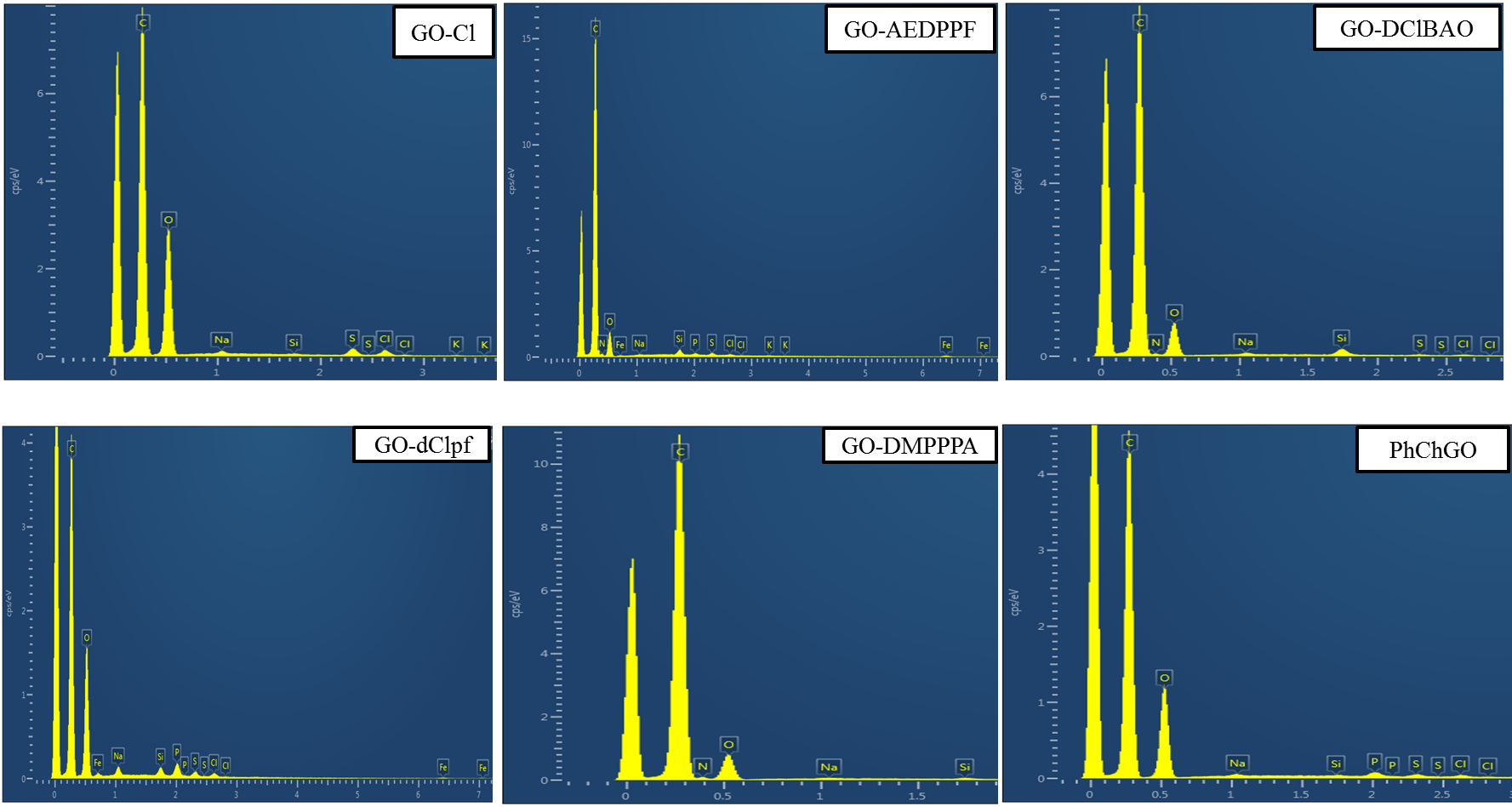


Figure 1SI. EDX spectra of GO modifications.


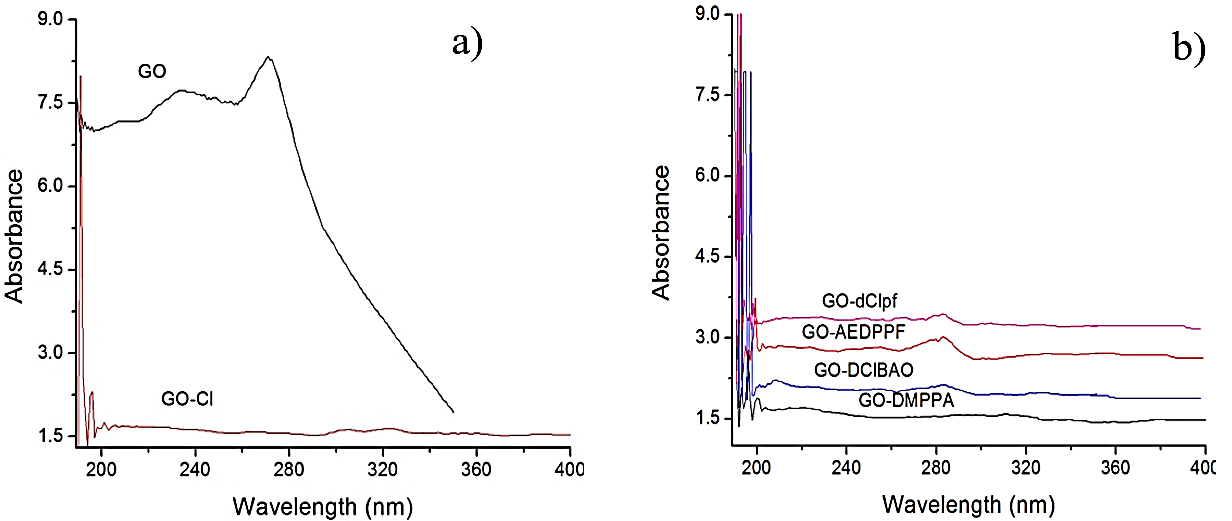


Figure 2SI. UV-Vis absorption spectra of GO and its modifications.





Figure 3SI. Thermogravimetric analysis results of modified GO samples. “GO iso” is the graphene oxide sample heated up to 125 ℃ and kept at this temperature for 30 minutes.


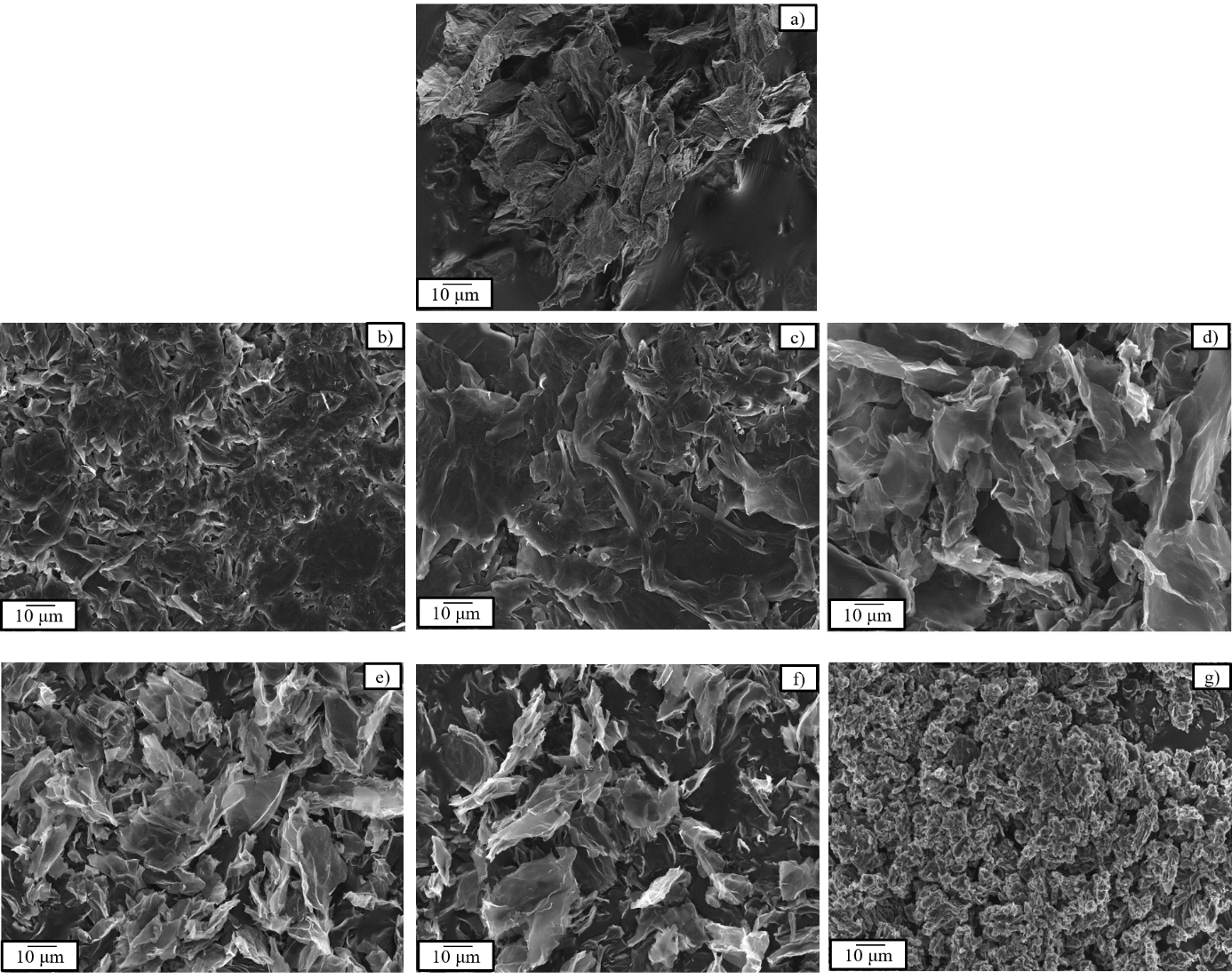


Figure 4SI. SEM images of: a) GO, b) GO-Cl, c) GO-DMPPA, d) GO-AEDPPF, e) GO-dClpf, f) GO-DClBAO and g) PhChGO.


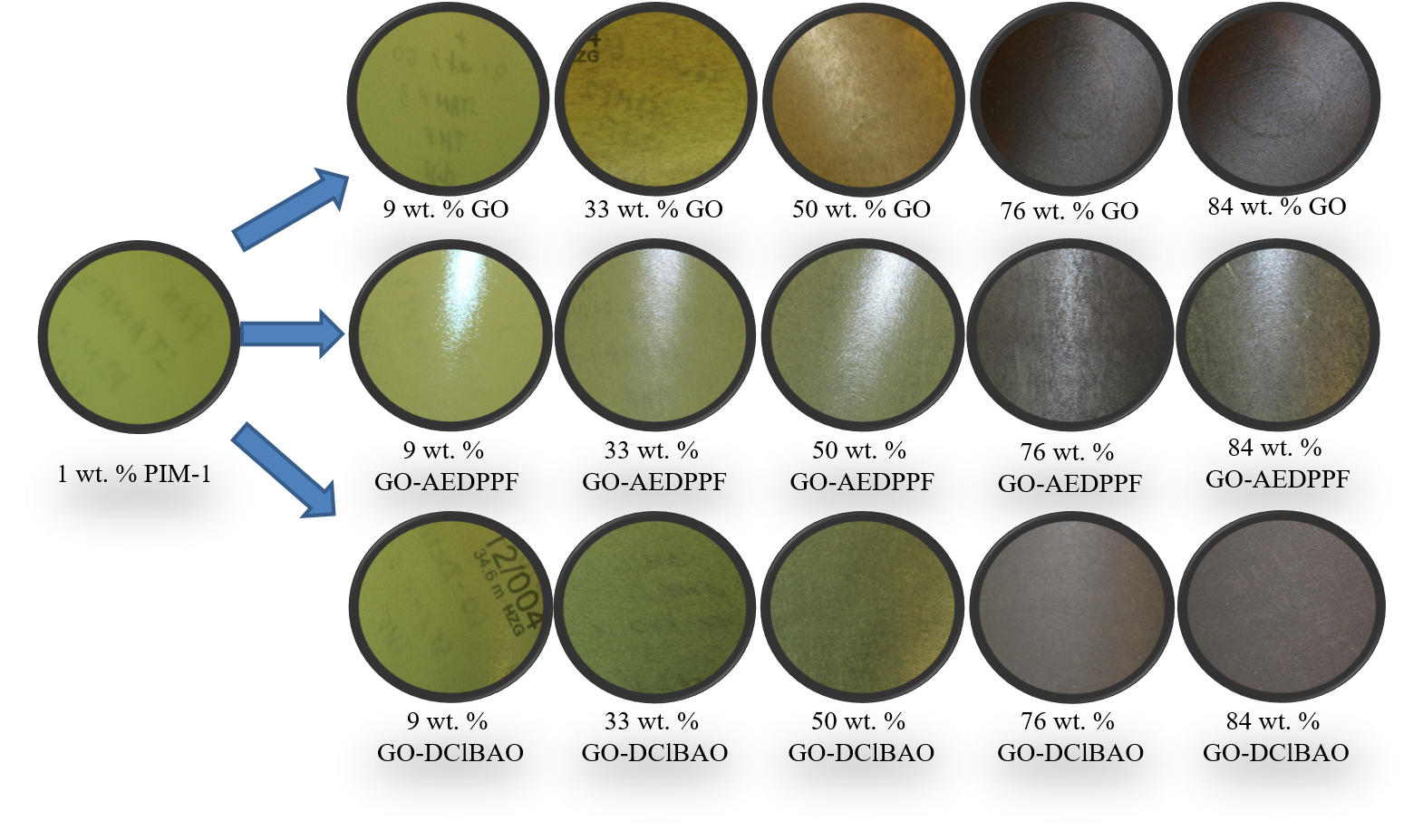


Figure 5SI. Optical images of PIM-1/GO (top), PIM-1/GO-AEDPPF (middle) and PIM-1/GO-DClBAO (bottom) thin film membranes on PAN.


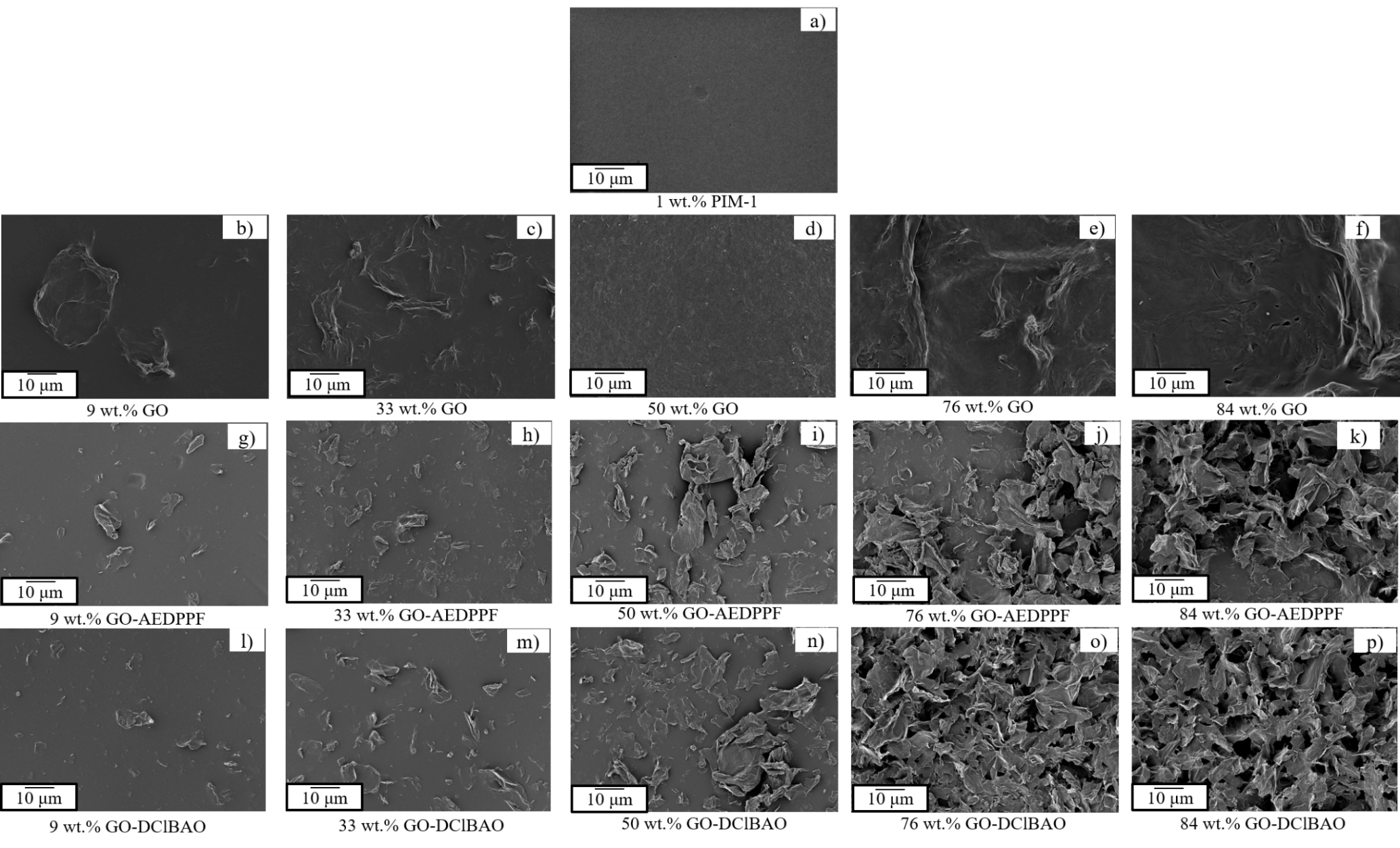


**Figure 6SI. SEM images of the surface of the TFC membranes: a) PIM-1; b-f) PIM-1/GO, g-k) PIM-1/GO-AEDPPF
and l-p) PIM-1/GO-DClBAO.**


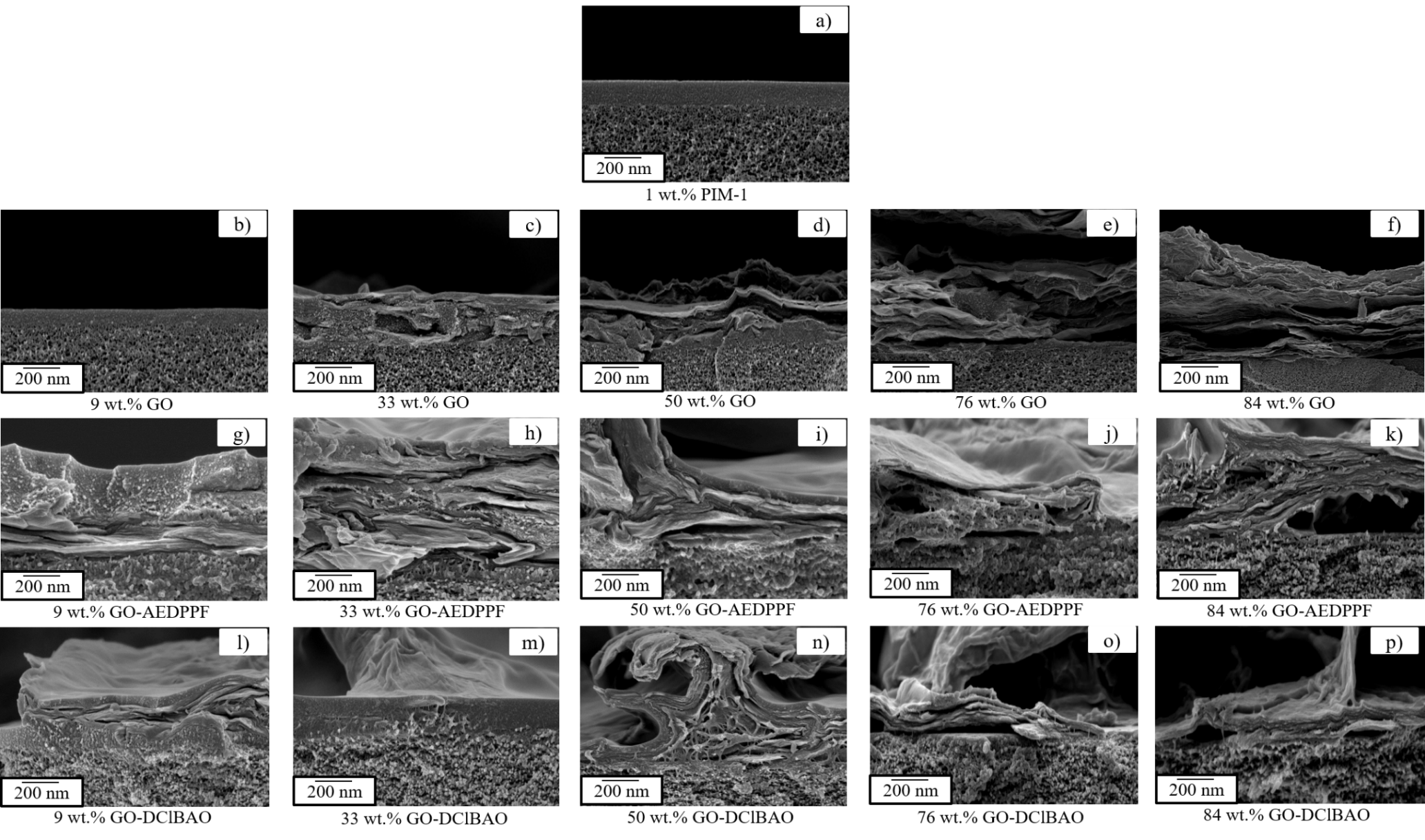


**Figure 7SI. Cross-sectional SEM images of the TFC membranes: a) PIM-1, b-f) PIM-1/GO, g-k) PIM-1/GO-AEDPPF and l-p) PIM-1/GO-DClBAO.**

Table 1SI. Permeance for the GO containing PIM-1 TFC membranes and the gas permeance comparison with the state-of the-art membranes
(data for all GO contents).

| Membrane  code | Filler | Wt.% of filler | Permeance, m^3^(STP)m^-2^ h^-1^ bar^-1^ | | | | Reference |
| --- | --- | --- | --- | --- | --- | --- | --- |
|  |  |  | N_2_ | CH_4_ | CO_2_ | O_2_ |  |
| PolyActive™ | - | - | 0.08 | 0.35 | 4.8 | 0.24 | (37) |
| Matrimid® 5218 | - | - | 0.0083 | 0.010 | 0.304 | 0.058 | (38) |
| Polyetherimide | - | - | 0.05 | 0.029 | 1.56 | 0.38 | (39) |
| PIM1-0.00096G^[[8]](#footnote-8)^ | Graphene | 0.00096 | 870 | 1450 | 12700 | 2260 | (1) |
| PIM-1 | - | - | 0.52 ± 0.16 | 0.83 ± 0.03 | 11.04 ± 0.34 | 1.69 ± 0.05 | **This work** |
| PIM1-0.1GO | GO | 9 | 0.42 ± 0.08 | 0.58 ± 0.12 | 7.32 ± 0.75 | 1.25 ± 0.12 |  |
| PIM1-0.5GO |  | 33 | 0.11 ± 0.01 | 0.16 ± 0.02 | 1.18 ± 0.63 | 0.24 ± 0.08 |  |
| PIM1-1GO |  | 50 | 0.07 ± 0.02 | 0.1 ± 0.03 | 0.1 ± 0.03 | 0.07 ± 0.02 |  |
| PIM1-3GO |  | 76 | 0.8 ± 0.57 | 1.08 ± 0.76 | 0.7 ± 0.5 | 0.74 ± 0.53 |  |
| PIM1-5GO |  | 84 | 1.3 ± 0.52 | 1.71 ± 0.68 | 1.43 ± 0.36 | 1.27 ± 0.48 |  |
| PIM1-0.1GO-AEDPPF | GO-AEDPPF | 9 | 0.18 ± 0.02 | 0.26 ± 0.03 | 4.55 ± 0.51 | 0.83 ± 0.1 |  |
| PIM1-0.5GO-AEDPPF |  | 33 | 0.17 ± 0.005 | 0.25 ± 0.007 | 3.3 ± 0.25 | 0.64 ± 0.03 |  |
| PIM1-1GO-AEDPPF |  | 50 | 0.83 ± 0.17 | 1.13 ± 0.23 | 3.43 ± 0.66 | 1.2 ± 0.23 |  |
| PIM1-3GO-AEDPPF |  | 76 | 30.98 ± 10.58 | 41 ± 14 | 26.85 ± 6.84 | 29.1 ± 9.58 |  |
| PIM1-5GO-AEDPPF |  | 84 | 63.58 ± 10.38 | 85.12 ± 14.39 | 49.13 ± 7.56 | 59.22 ± 9.45 |  |
| PIM1-0.1GO-DClBAO | GO-DClBAO | 9 | 0.38 ± 0.01 | 0.61 ± 0.02 | 8.17 ± 0.25 | 1.41 ± 0.05 |  |
| PIM1-0.5GO-DClBAO |  | 33 | 0.39 ± 0.02 | 0.62 ± 0.04 | 8.16 ± 0.3 | 1.41 ± 0.07 |  |
| PIM1-1GO-DClBAO |  | 50 | 2.11 ± 0.57 | 2.85 ± 0.65 | 5.74 ± 1.07 | 2.52 ± 0.51 |  |
| PIM1-3GO-DClBAO |  | 76 | 103.77 ± 11.62 | 141 ± 23.44 | 75.42 ± 7.59 | 96.1 ± 13.34 |  |
| PIM1-5GO-DClBAO |  | 84 | 127.52 ± 2.85 | 176.65 ± 2.5 | 97.22 ± 0.93 | 118.74 ± 3.23 |  |

**
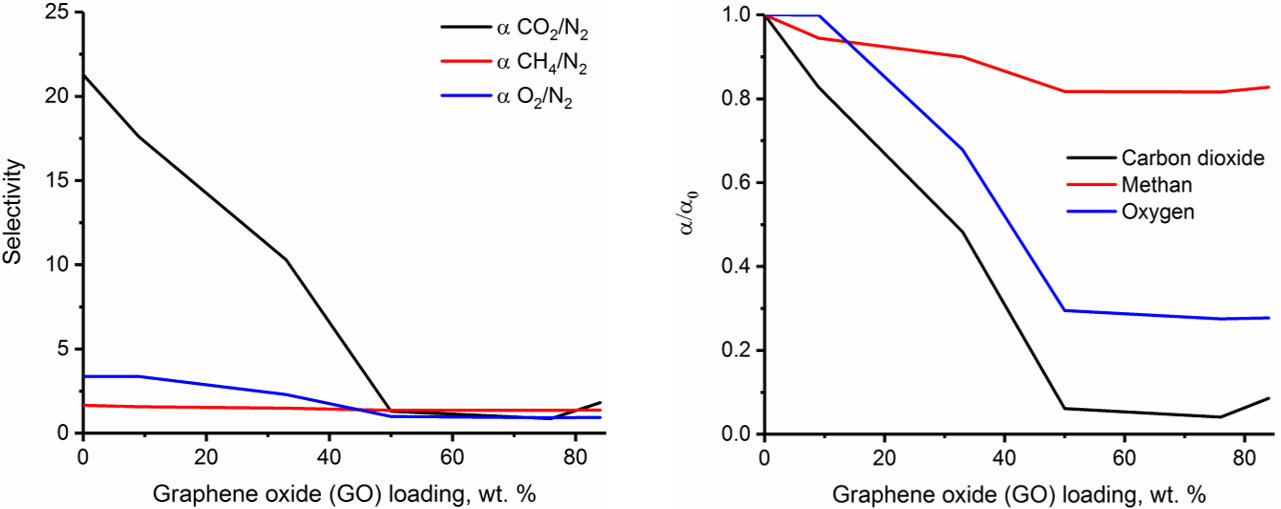
**

**Figure 8SI. GO containing PIM-1 thin film composite membrane's selectivity for gas pairs.**


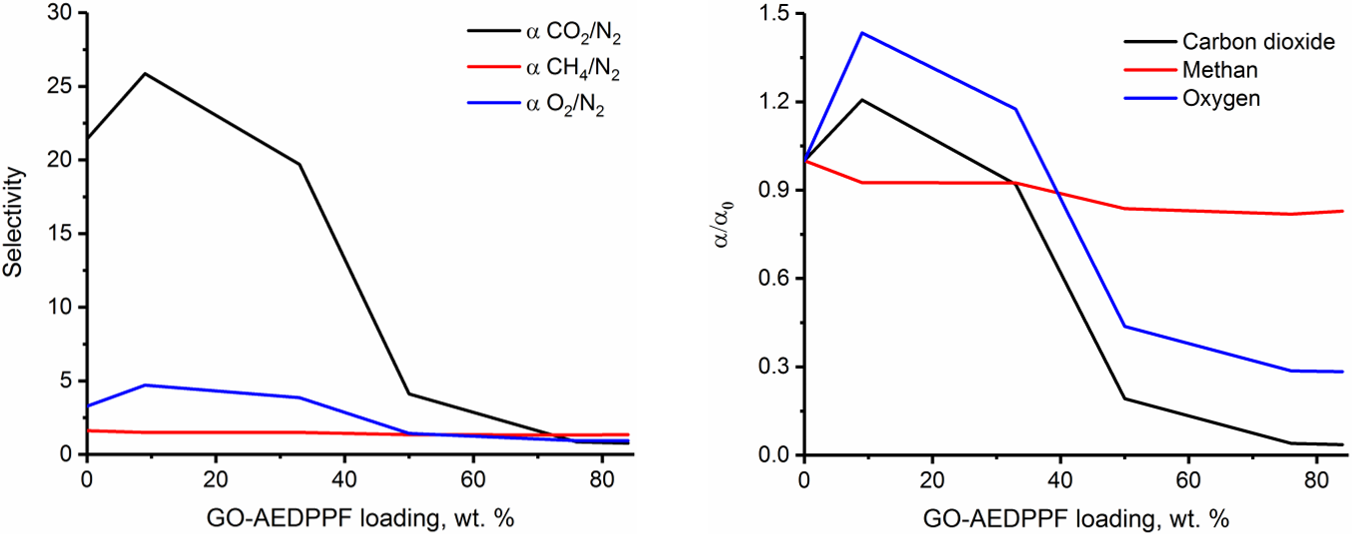


Figure 9SI. GO-AEDPPF containing PIM-1 thin film composite membrane's selectivity for gas pairs.


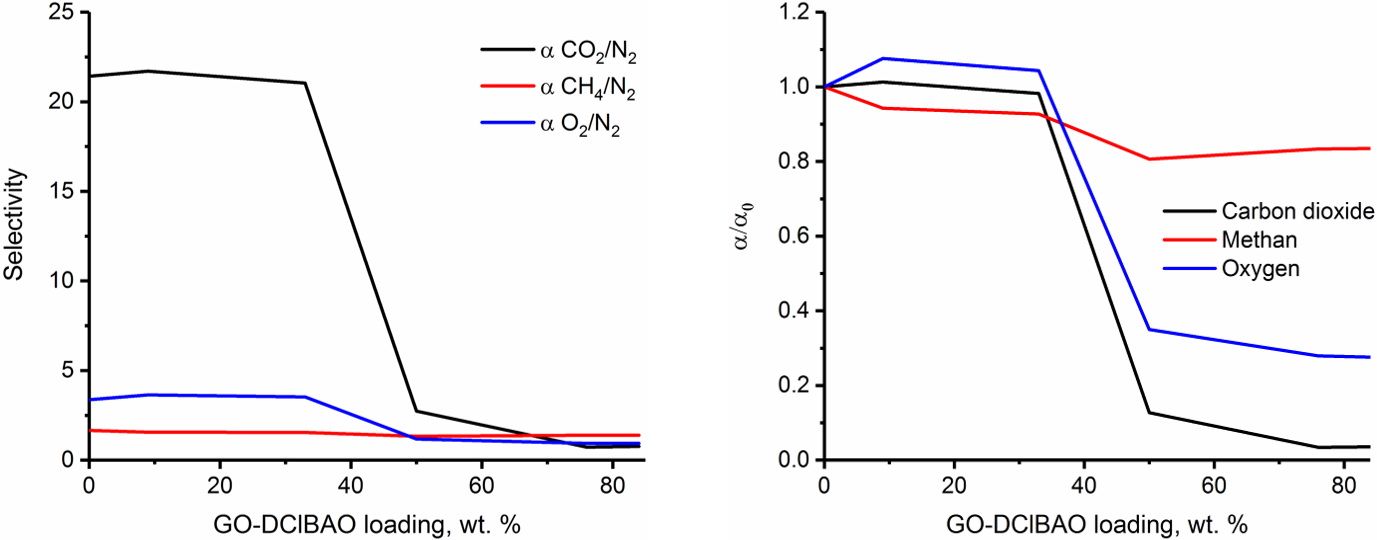


Figure 10SI. GO-DClBAO containing PIM-1 thin film composite membrane's selectivity for gas pairs

**Table 2SI. Permeance and selectivity of PAN porous membrane used as a porous support for formation of TFC membranes.**

| Gas | MM,  g mol^-1^ | Permeance,  m^3^(STP)m^-2^ h^-1^ bar^-1^ |
| --- | --- | --- |
| CH_4_ | 16 | 264.4 ± 2.3 |
| N_2_ | 28 | 200 ± 3.0 |
| O_2_ | 32 | 186.9 ± 0.8 |
| CO_2_ | 44 | 158.5 ± 0.3 |
| Ideal selectivity α_ij_ | | |
| O_2_/N_2_ | 16 | 0.93 ± 0.02 |
| CH_4_/N_2_ | 28 | 1.31 ± 0.03 |
| CO_2_/N_2_ | 32 | 0.78 ± 0.01 |
| CO_2_/CH_4_ | 44 | 0.59 ± 0.01 |

Polyacrylonitrile (PAN) membrane shows gas transport properties correlating with the Knudsen type transport in porous media. The equation SI1 [4] describes dependence of the gas flux through the porous membrane as dependent on the reciprocal square root of the molecular weight of the penetrant:

$$J_{K}=\frac{2r}{3}\sqrt{\frac{8R_{g}T}{\pi M}\cdot} \frac{\Delta n}{L} (SI1)$$

where *r* – radii of gas molecule, *R_g_* – the ideal gas constant, *T* – the temperature, *M* – molecular weight of gas, *n* – molecule density (molecule · m^-3^ ), *L* – the gas permeance





**Figure 11SI. Justification of the Knudsen type gas transport in PAN porous membrane used as support for TFC membranes preparation.**

1. 1 *Helmholtz-Zentrum Geesthacht, Institute of Polymer Research, Max-Planck-Str. 1, 21502 Geesthacht, Germany* [↑](#footnote-ref-1)
2. 2 *Baku State University, Z. Khalilov str. 23, AZ 1148, Baku, Azerbaijan* [↑](#footnote-ref-2)
3. [↑](#footnote-ref-3)
4. [↑](#footnote-ref-4)
5. [↑](#footnote-ref-5)
6. [↑](#footnote-ref-6)
7. Corresponding author.

   *Tel:* +49-41-5287-2425. *E-mail address:* volkan.filiz@hzg.de [↑](#footnote-ref-7)
8. For the gas permeability experiments of the PIM1-0.00096G membrane, the thick polymer film was used and the permeability is shown in barrer. [↑](#footnote-ref-8)
